# Examining confidential wholesale margin estimates in European countries for the price negotiation of patented drugs in Germany: a statistical model

**DOI:** 10.1186/s13561-024-00503-9

**Published:** 2024-04-12

**Authors:** Iris an der Heiden, Guido Schiffhorst, Laura Müller, Martin Albrecht, Arne Bartol, Stefanie Wiberny

**Affiliations:** 1 IGES Institute GmbH, Berlin, Germany; 2 Health Economics, Market Access & Reimbursement, Janssen-Cilag GmbH, Pharmaceutical Companies of Johnson & Johnson, Neuss, Germany

**Keywords:** Drugs, Wholesale margins, International reference pricing, External reference pricing, Ex-factory prices, European pharmaceutical prices, AMNOG, Benefit assessment, Price negotiation, Estimation method, Statistical model

## Abstract

**Background:**

Based on the legal framework laid down in section 130b (9) of Book V of the German Social Code, various criteria are relevant for the negotiated price for new patented drugs in Germany. European reference prices (ERPs) are one criterion. The ERP is based on the ex-factory prices (EFPs) of the countries included in the European country basket. However, in some of these countries, the EFP is not published due to confidential wholesale margins. Wholesale margins must therefore be estimated and deducted from purchase prices. In this context literature-based estimates to date do not assume regressive margins with higher pharmaceutical prices. This assumption is questionable and can lead to systematically underestimated country prices, especially for high-priced drugs. Percentage wholesale margins in the majority of European countries develop to a comparable extent regressively with increasing prices. It should therefore be examined (1) whether statistical models can predict the margins of individual countries, in principle and especially for countries where margins are unknown and regressive trends are likely, and (2) to what extent the estimation of margins improves when regressive statistical models are used to estimate margins instead of cross-price averages published in the literature.

**Methods:**

Qualitative preliminary research explores the basic wholesale pricing mechanisms in countries with confidential wholesale margins. Wholesale margins for reimbursable drugs were then modeled for regulated European countries. Estimation quality and impact of the model was compared to estimations based on average margins.

**Results:**

In both regulated countries *and* in countries with confidential wholesale margins, percentage margins of wholesalers develop regressively as drug prices rise. Regressive courses of margins can be resiliently modeled for the regulated countries using a power distribution with significantly lower mean squared errors in a linear mixed model in comparison to literature-based estimations with country-specific cross-price averages.

**Conclusion:**

If there is reason to believe that margins are regressive, confidential wholesale margins are expected to be better estimated by the power function based on margins of regulated countries than by the published country-specific average margins, reducing significantly inaccurate effects on margin estimations of high-price drugs.

**Supplementary Information:**

The online version contains supplementary material available at 10.1186/s13561-024-00503-9.

## Background

As a result of the German Act to Reorganize the Pharmaceuticals’ Market in the Statutory Health Insurance System (AMNOG) introduced in 2011, prices for new patented drugs are determined on the basis of an additional benefit assessment [1]. The AMNOG process is therefore divided into two parts: the additional benefit assessment and the price negotiation [2]. In the price negotiation, a reimbursement amount is negotiated between the Association of Statutory Health Insurers (SHI) and the pharmaceutical company concerned. The legislator has established criteria for the price negotiation, which are specified in a framework agreement between the SHI and pharmaceutical manufacturers [3]. For the negotiation of a reimbursement amount, (I) the additional benefit as a result of the early benefit assessment, (II) the annual therapy costs of the established appropriate comparative therapy, (III) the annual therapy costs of comparable drugs within the authorized indication(s), (IV) the actual dispensing prices in other European countries and (V) the negotiated quantities and durations are of decisive importance. The price criterion ‘dispensing prices in other European countries’ of a country basket, abbreviated as European Union (EU) reference prices below, involves some methodological and conceptual challenges.

Referring to the framework agreement, the pharmaceutical manufacturer needs to submit the real net prices of the 14^1^ European reference countries—which can be understood as ex-factory price (EFP) reduced by mandatory and possible confidential rebates—to the SHI [3]. While EFPs are officially published in most of the countries of the country basket, they are only published on pharmacy purchase price level in Denmark, Finland, Sweden and the Netherlands. As the margins are confidentially negotiated between manufacturer and wholesaler in these countries, they need to be estimated.^2^ This estimation of confidential wholesale margins is a topic that is heavily discussed within the price negotiations between pharmaceutical manufacturer and SHI, as the estimated numbers vary extremely on both sides. Therefore, this paper discusses different estimation methods and aims to identify methods with better estimation quality. This is done by reviewing the corresponding literature on current estimation data, conducting qualitative expert interviews as well as modeling wholesaler margins based on the official published regulations in other European countries and their impact on price negotiations.

## Literature review

In the international comparative literature, wholesale margins^3^ are mainly described in terms of price differentiation principles and corresponding distribution structures in the individual countries. The focus here is usually on the efficiency of wholesalers (with regard to providing a full range of products and to ensuring nationwide drug supply, in particular). It is discussed whether the remuneration of the wholesale trade is generally sufficient against the background of increasing quality requirements for distribution and in view of shifts in the drug price mix [5].

The literature dealing with wholesale pricing is dominated by the question of the determinants and appropriateness of the average yield level. Among the determinants of the earnings situation in wholesaling investigated in the literature are the share of direct distribution by pharmaceutical manufacturers and product mix of direct distribution, cost recovery of margins across the entire price spectrum with regard to the distributed quantities in the different price segments or margin design in the different product and price segments in the context of a mixed calculation. In addition, this includes the yield in sub-segments of the market in case of different pricing systems (for example, for the reimbursement market versus self-payer market or for generics), as well as discounts and added value services of the pharmaceutical manufacturers’ wholesalers [6, 7]. Regarding costs, literature refers to cost differences of distribution in urban and rural regions or opportunities for increasing efficiency in storage and distribution as well as the competitive situation, for example [6].

In European countries, the design of wholesale margins varies in line with structural differences. Wholesale margins are based on the ‘solidarity principle’, which covers cross-subsidization between product types [6]. In regulated countries with transparent wholesale margins, there are differentiations depending on the price level because the costs of distributing drugs do not increase in line with the price of the drug. This means that for drugs with very low prices, percentage wholesale margins of more than 100% may be justified in order to enable a cost-covering supply. At the same time, margins can also regress below 1% with increasing price levels in relation to the EFP. Current arrangements in the countries are percentage fixed, percentage regressive or capped margins as well as combinations of these [5, 6]. Exceptions with regard to regressive margins are Italy and Ireland, which have a linear percentage margin. In regard to Italy, Walter et al. point out that in some countries, especially in southern Europe, high-priced drugs are only available through the hospital so that wholesale is not involved. In Italy, the market portion of the drugs in hospitals is particularly high [5]. Therefore, there is little need for a legally stipulated regression of wholesale margins for higher-priced pharmaceuticals. In Ireland, there is a regulation referring to a fixed margin. Nevertheless, negotiations between wholesalers and pharmacists regarding the margin split are possible [8].

There are few systematic or analytical international comparisons of wholesale margins in the literature that affect margins. Presumably, this is due to the large number of structural differences across countries, in particular. The best that can be found in the literature is descriptive accounts of margins, usually published in combination with other price components of the overall supply chain [9]. There are no scientific models that examine the impact of tiering or capping margins on overall drug prices or drug supply. It is simply noted that missing absolute minimum margins or absolute maximum margins calculated too low can endanger the business models of wholesalers if there are, for example, changes in quality requirements or if the number of very low-priced or high-priced drugs grows disproportionately [5].

The estimation of confidential wholesale margins in the pharmaceutical market has not yet been the subject of scientific consideration, either. Rather, it is a practical necessity in the price negotiation process for the calculation of the EU reference price. International (external) reference pricing in price negotiations is discussed in the scientific literature mainly with regard to its effect on drug price regulation. In this context, further effects of reference pricing on the pharmaceutical market are investigated. These include innovation incentives or the country order of market entries [10, 11]. In this context, the literature also discusses problems of determining an appropriate EU average, e.g. due to the lack of comparable data [12]. Methodological or data-related problems of an estimate of confidential wholesale margins for individual countries, which is part of the calculation of the EU average price, are not explicitly addressed. Only rarely is this directly referred to (e.g. “In these countries with no statutorily regulated wholesale remuneration, ex-factory prices can, at best, be calculated on the basis of an estimated average wholesale margin.” ([7], p. 354). In Austria and Switzerland, regulations on the procedure of the price commissions for the determination of the EU average price explicitly stipulate that the EFP in the countries with confidential wholesale margins is to be determined by deducting average margins [13, 14]. The price level-independent average margins stated are named without source citation but can – with the exception of the values for the Netherlands – be traced back to a single publication, which can also be viewed as outdated given the dynamically developing market for pharmaceutical products. In particular, this is Kanavos et al. [6], who published an overview in 2011 (“Cost-containment policies in public pharmaceutical spending in the EU”) based on published country profiles of wholesale associations with aggregate data for member wholesalers. This includes average margins for both regulated countries and countries with confidential wholesale margins: Denmark, Finland, Sweden and the Netherlands. Based on this data from Kanavos et al. [6], Carone et al. [15] pick up the ranges mentioned and show average values for them. Table 1 summarizes the published values of average wholesale margins in Europe. In summary, in the absence of alternatives, at least comprehensible in the literature up to 2021, the estimation of confidential margins is based on outdated industry compilations from 2011.

**Table 1:** Published average wholesale margins for countries in the country basket with confidential margins

**Source**	**Countries**			
	**Denmark**	**Finland**	**Netherlands**	**Sweden**
Kanavos et al. [6]	6–7%	3%	13–24%	2–3%
Carone et al. [15]^a^	6.5%	3%	18%	2.5%
Swiss Regulation [14]	6.5%	3%	6.5%	2.7%
Austrian Price Commission [13]	6.5%	3%	6.5%	2.9%

Authors’ additions: Average, when range of margins was provided by Kanavos et al. [5]

Beyond Kanavos’ compilation [6], information on average margins can only be found irregularly in scientific or specialist literature for individual European countries and rarely in a comparative overview. The European Federation of Pharmaceutical Industries and Associations (EFPIA) regularly publishes an aggregate average margin for all European member countries (e.g. 4.9% in the 2019 report) but does not present the average values for individual countries [9]. These and similar compilations are mainly based on country reports that refer to associations of wholesalers that receive data on average profit margins from their member companies and aggregate these to a value for the entire country [6]. Data on the statistical distributions underlying these averages are not available. This means that no statement can be made as to whether using the average values for margin estimation for drugs at different price levels leads to systematic distortions. The fact that both wholesalers and pharmaceutical enterprises in the respective countries have a high interest in maintaining the confidentiality of agreed wholesale margins may be one reason why differentiated data on reimbursement market margins are not available in the literature and why no survey methods for estimating these margins are presented. The literature does not contain a description of the price-setting mechanisms of wholesalers in countries with confidential margins.

**Fig. 1:**
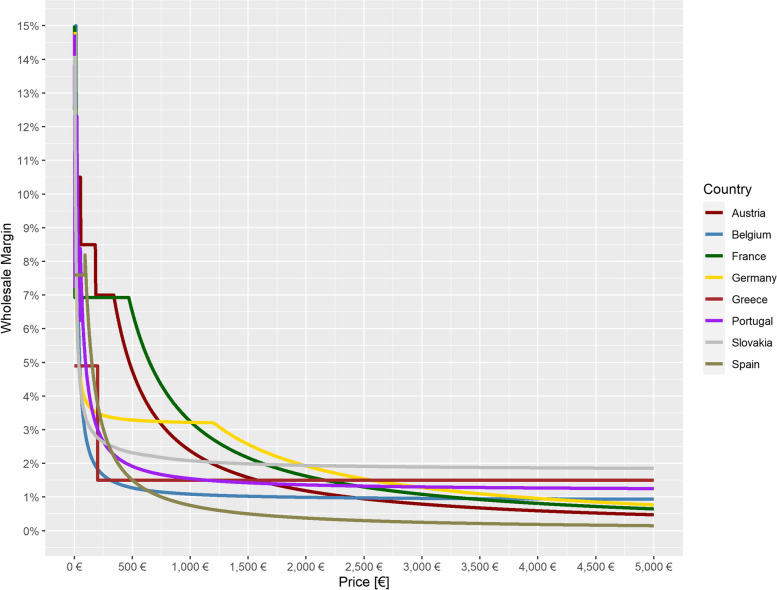
Officially regulated wholesale margins by price for selected European countries

In the majority of the countries of the country basket [3], wholesale margins are transparently regulated by law and show a regressive trend which is visualized graphically in Fig. 1. This means that for Austria, Belgium, France, Germany, Greece, Portugal, Slovakia and Spain, all data are available to determine wholesale margins for different EFPs. An overview of European country-specific applicable statutory margin regulations for reimbursable medicines [16–23] is provided (see Additional file 1). The regressive course is the result of different regulatory approaches in some cases, leading to more or less flowing or graduated courses.

## Research question

**Fig. 2:**
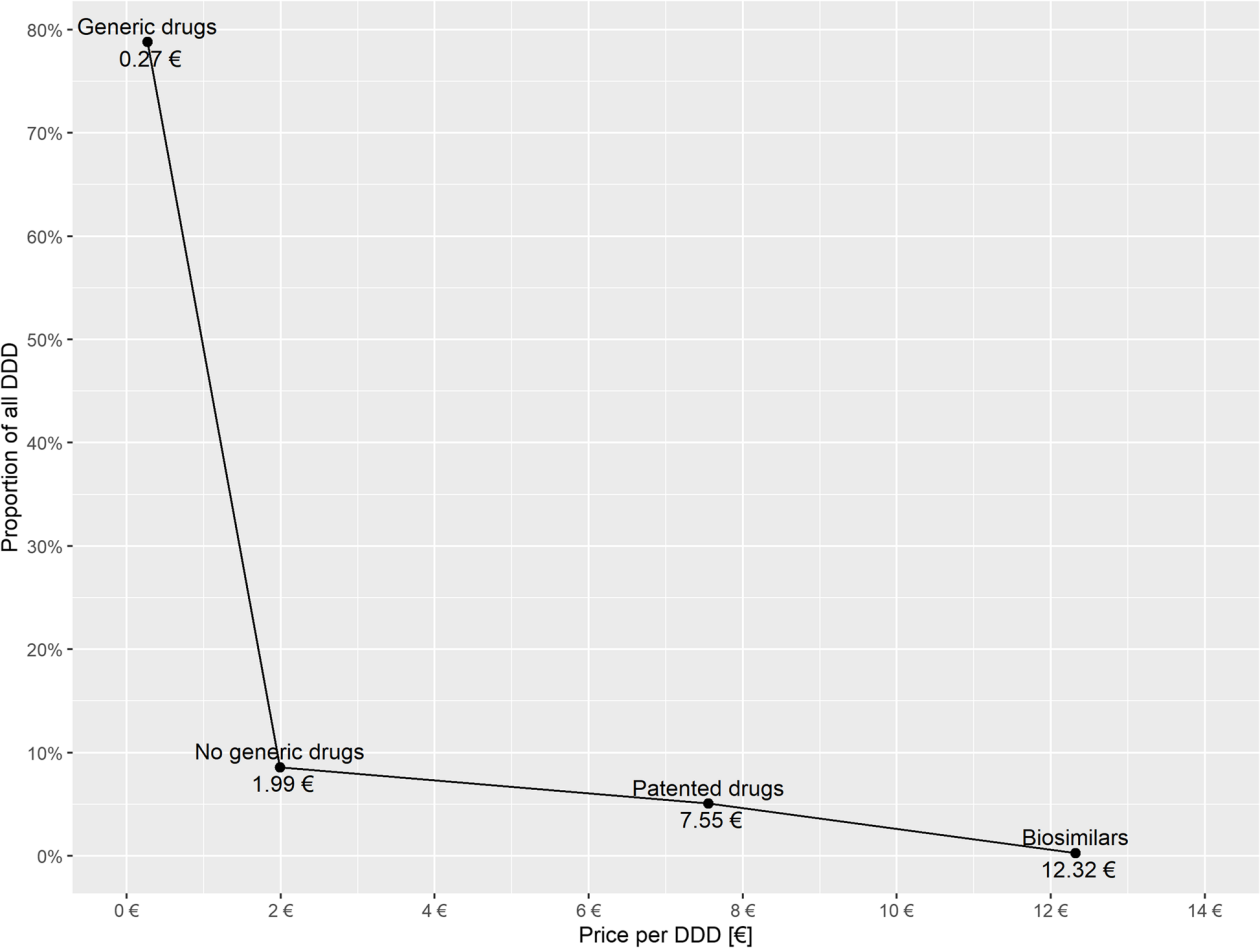
Average prices and volumes of DDD by drug segment (reimbursement market Germany 2020) (National Prescription Information (Data source: Insight Health))

**Fig. 3:**
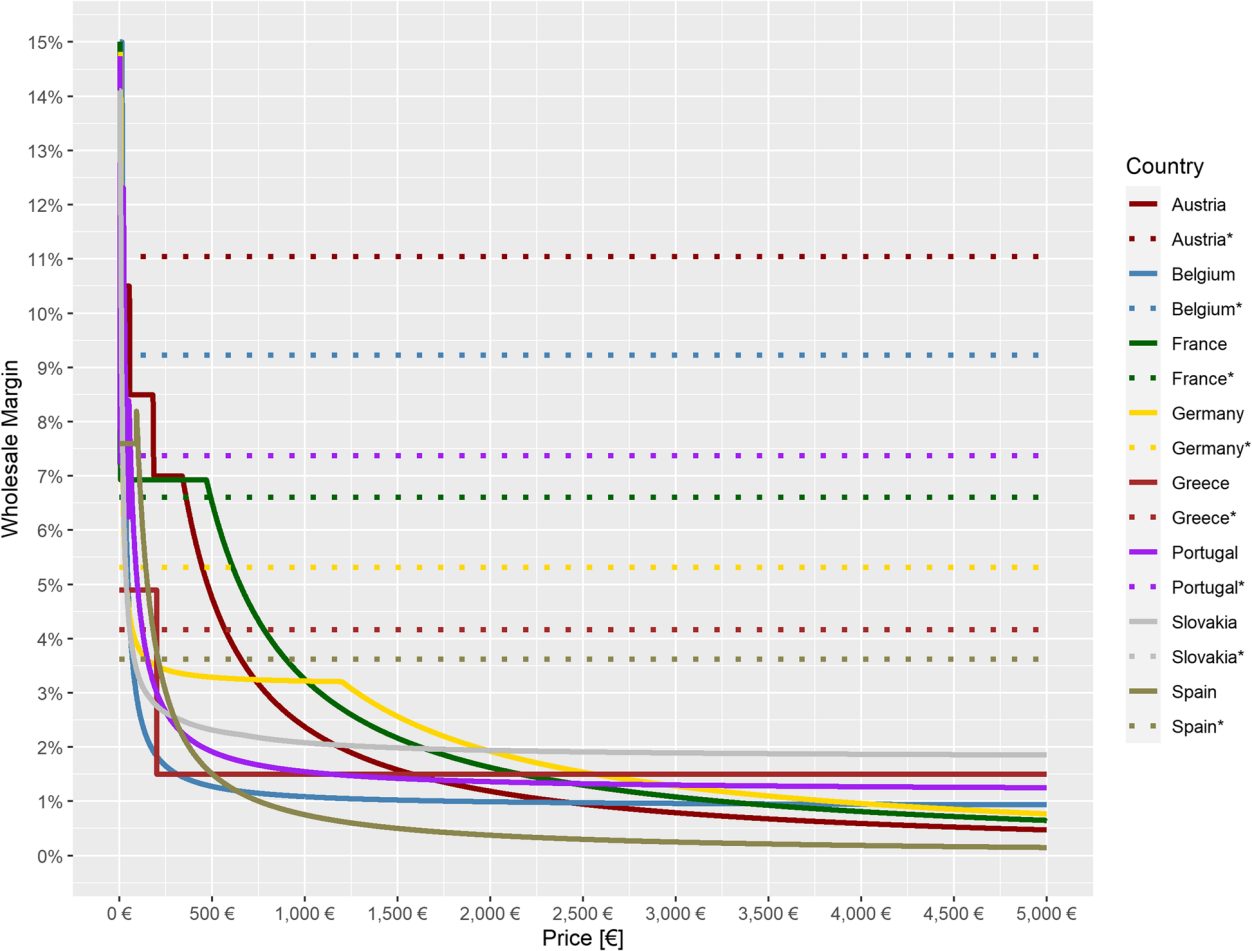
Comparison of the regulated margins with the average margins from the literature [6] by country. *average margin

Assuming that even in countries with confidential wholesale margins, the margin trend may take a regressive form as the drug price increases, using literature-based averages to estimate wholesale margins for specific drugs does not produce realistic results. This is mainly caused by the following conditions:

1. Highly different quantities of drugs in the price segments correspond to a left-skewed distribution function [5], for which averaging is not appropriate.
  While the regulated margin can be calculated independently of the quantities prescribed in a country, the average margin inevitably includes quantity effects. Figure 2 shows the quantity distribution of prescribed drugs (in defined daily doses (DDD)) for the reimbursement market in Germany as a function of different price levels (average prices per DDD). As the quantity of products is higher in the lower price segment, the volume distribution as a function of the price level, which is shown in Fig. 2, is strongly left-skewed. This explains why the average margin is quite high but does not represent the entire distribution. Additionally, the regressive margin curve intersects the average margin curve at a comparatively low price in Fig. 3, indicating that volumes are strongly concentrated in the low price range.
2. The average margins presented in the literature probably refer to the total sales of the wholesalers, thus also including the non-reimbursement market, while the ERPs, for which the average margins are used as estimates, are only needed for the price determination in the reimbursement market.
3. In particular for high-priced patented drugs, the assumption of average margins independent of price level results in a systematic overestimation of margins and thus an underestimation of the drug price in countries with confidential wholesale margins.
  The following example illustrates that the use of average values as wholesale margin estimates for a specific drug can lead to considerable distortion if regressive margins actually apply. According to Carone et al. [15], the average wholesale margin for Germany is 5% of the pharmacy purchase price (PPP). If this 5% was used as an estimate for a drug with a PPP of €3,000, the wholesale margin would be €3,000 * 5% = €150. In reality, it is capped at €38.50 in Germany and therefore corresponds to only €38.50/3,000 = 1.3%. A similar bias can consequently be assumed if the average values given in Kanavos [6] are taken as estimates for Denmark, Finland, Sweden and the Netherlands, without taking into account a possibly regressive margin trend. The use of average sizes as estimates for confidential wholesale margins may then lead to significantly distorted results for high-priced pharmaceuticals.

Against this background, and taking into account that the question of an appropriate basis for estimating confidential wholesale margins has not yet been reflected in the academic literature, the following key research questions arise:

1. Are there any reliable indications that in countries with confidential wholesale margins, margins tend to decline as drug prices increase?
2. How can wholesale margins be estimated using models that account for a regressive margin trend?
3. For countries with confidential margins: To what extent can a regressive model improve estimation accuracy compared to price-independent average margins?
4. What impact would the estimate of wholesale margins have on ERPs?

## Methods

Research Question (1) can be answered using qualitative methods to complement the information about margin formation mechanisms reported in the literature. The relevant countries predominantly have a structure with only a few wholesalers who also cover the entire price spectrum of pharmaceuticals. In initially confidential expert interviews conducted by the co-authors from the IGES institute with representatives of these wholesalers or their permanent contractual partners on the side of the pharmaceutical manufacturers, the extent to which margins are negotiated on the basis of price level was investigated. The interviews were transcribed and summarized in regard to the interview guideline respective the core questions (see Additional file 2).

Research Question (2) is answered by statistical modeling, which analyzes the data of the regulated countries in the EU basket with regard to underlying, statistically describable patterns. The official margin-regulation in the majority of EU countries shows that there is a dependency between price level and wholesaler-margin in percent. For EFPs of €1 to €5,000 per country, margins can be calculated for the regulated countries that correspond to the ‘empirical’ values resulting from the legal requirements and compared to the average values published in the literature.

To answer Research Question (3), a descriptive comparison first shows the differences between the two estimation methods, i.e. the statistical model and using uniform average margins. In a second step, an attempt is made to find a model for the regressive distributions of the regulated countries that can form an alternative basis for estimating wholesale margins and that can describe the dependence of the margin on the price level. Afterwards, both approaches, estimation by the identified model and by average margins, are compared and evaluated by Mean Squared Error (MSE) for the countries with known margins. The analysis is conducted using R 4.1.2 (Bird Hippie) and package *lme4*.

Finally, in regard to Research Question (4), the estimation model is applied to the countries with confidential margins and an impact analysis is performed to determine the hypothetical effect at the level of negotiated drug prices by means of concrete cases and scenarios on the impact of the different estimation methods on ERPs.

## Results

### Expert interviews

The expert interviews were conducted in July and October 2022. For this purpose, two wholesaler contacts and one pharmaceutical manufacturer were obtained, covering the four countries with confidential wholesale margins: Wholesaler Oriola for Sweden and Finland, one of the two wholesalers in Denmark, Tjellesen Max Jenne A/S, and the pharmaceutical manufacturer Janssen-Cilag, the Netherlands. The pharmaceutical manufacturer Janssen-Cilag also has a contractual relationship with the Dutch wholesalers for medicines in different price ranges and is therefore an equal interview partner on the mechanisms of negotiating margins between wholesalers and pharmaceutical manufacturers.

As result, the interviewees of the wholesalers were able to confirm, without disclosing the confidentiality of the negotiations, that (I) the wholesalers in the Northern European countries supply the complete price range and that (II) the margins negotiated regress as prices increase. Even though there are sometimes higher costs for infrequently requested expensive drugs compared to frequently requested inexpensive drugs, these effects on margins are low as compared to the overall effects that price differences have on the percentage margins. Since the margins are negotiated separately and confidentially with the individual drug manufacturers, it is possible that for manufacturers whose products are at a similar price level, fixed margins are negotiated for all products of this manufacturer. Furthermore, some other factors may be taken into account, such as order quantities per price (‘Order Line Value’) or requirements of special handling relevant to expenses (cooling needs, fragility, etc.). A minimum price may additionally be set if cost-covering operation so requires. However, all that does not fundamentally change the principle of different, staggered margins for various price categories, which are systematically used by wholesalers in price negotiations. In conclusion, the interviews of the wholesalers confirm the hypothesis that (also in countries with confidential wholesale margins) it is reasonable to assume a regressive margin function. In addition, the interviewee from the pharmaceutical manufacturer for the Netherlands stated that service contracts are concluded with wholesalers that include two different pricing models: Distribution of low-cost drugs is remunerated with a fixed percentage of the drug price for each package, and distribution of high-cost drugs is remunerated with a fixed absolute fee. The cut-off point between low-cost and high-cost drugs is confidential and is redefined on a regular basis. Accordingly, the development of the percentage margins with increasing price also shows a regressive course for the Netherlands: The percentage margin remains constant up to a certain margin and decreases regressively with 1/x from a cutoff point. Thus, it can be assumed for all countries with confidential wholesale margins that they apply - at least in significant parts of distribution - regressive margin curves as the price of drugs increases.

### Statistical model

In a first step, the selection of the countries which are included in the statistical model is defined. Based on the interviews with wholesalers, it can be concluded that in countries with confidential margins the confidentially negotiated margins also follow a regressive curve as it was confirmed that price levels play a significant role within the negotiations. Therefore the model is also based on countries with official regressive margins regulation. In consequence countries with regulated but fixed margins, i.e. Italy and Ireland, are excluded from the model. Since wholesale margins in the Czech Republic are not clearly regulated or there is only one common margin for pharmacists and wholesalers [6], which is divided between them in negotiations, this country is likewise also not considered further here. Finally, the model is based on the following eight countries: Austria, Belgium, France, Germany, Greece, Portugal, Slovakia and Spain. Figure 3 shows for the regulated countries both the literature-based uniform average margins and the margins differentiated for different drug prices (EFP) according to the country-specific legal regulations for wholesale. It is shown that uniform average margins underestimate margins in only some cases, when drug prices are very low, but overestimate margins in most of the drug price range, in some cases significantly.

In the search for a distribution form that describes the courses of wholesale margins across all countries, the power distribution proves to be suitable, since linear relationships of the form

1 $${log}_{10}\left({margin}_{i}\right) = {log}_{10}\left({a}_{i}\right)+ {b}_{i}*{log}_{10}\left(price\right)$$

are identified between logarithmized wholesale margins and prices in relevant price ranges in each country.

The regression equation is used to formulate a linear mixed model for ${log}_{10}\left({margin}_{i}\right)$ using ${log}_{10}\left({price}_{i}\right)$ as independent variable and allowing each country to have an individual intercept ${log}_{10}\left({a}_{i}\right)$ and slope ${b}_{i}$ by means of random effects. Country-specific point estimates for ${a}_{i}$ and ${b}_{i}$ are derived and substituted into the power distribution formula ${margin}_{i}={a}_{i}*{price}_{i}^{{b}_{i}}$ to obtain estimated values for the country-specific wholesale margins. Overall wholesale margins are determined by averaging over the countries.

For the overall margins, a 95% prediction band is determined using naïve bootstrap. 999 samples with replacement are drawn for each price and the 2.5 and 97.5 percentiles serve as a prediction band.

#### Power distribution

**Fig. 4:**
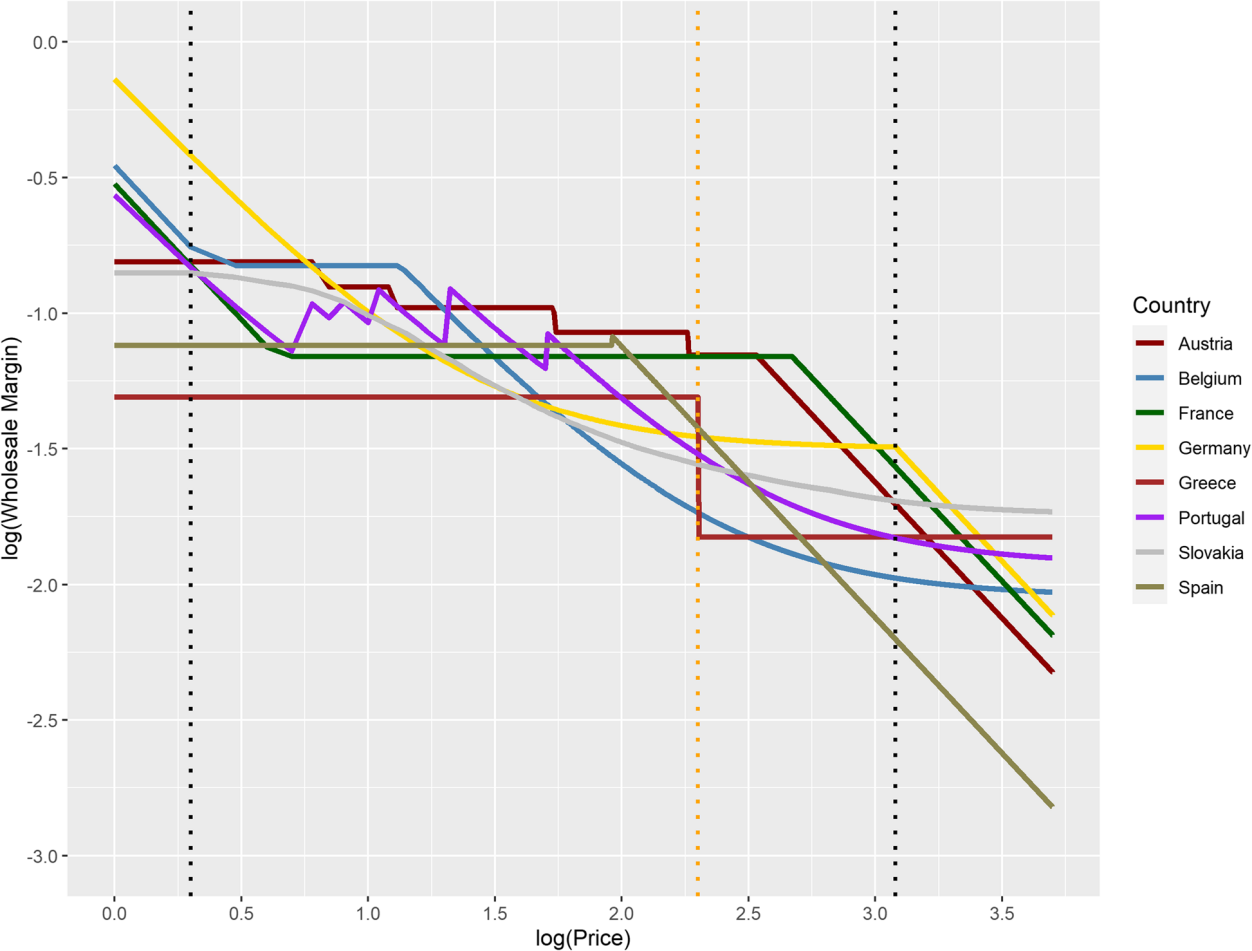
Visual verification of the appropriateness of the power distribution *log(margin)* ~ *log(price)*

In order to investigate whether the power distribution is suitable to describe the wholesale margins, Eq. (1) was applied to country-specific margins depicted in Fig. 3. Figure 4 shows the results.

Roughly four price ranges can be distinguished visually. Up to ${log}_{10}\left({price}_i\right)=0.3\left(\approx \EUR 2\right)$, there are linear relationships between log prices and log margins. Germany, Belgium, France and Portugal show the same slope starting from different levels, i.e. the exponent ${b}_{i}$ of the country-specific power distributions for this price range is the same in the four countries. The other four countries have constant margins in this price range, which is shown as horizontal lines with ${b}_{i}=0$ in the logarithmic plot. A comparable picture emerges for the range from ${log}_{10}\left({price}_{i}\right)=3.1 \left(\approx \EUR \mathrm{1,200}\right)$ with other countries showing equal exponents ${b}_{i}$.

Up to ${log}_{10}\left({price}_{i}\right)=2.3 \left(\approx \EUR 200\right)$, the wholesale margins show a very heterogeneous picture. The wholesale margins of Greece continue to be constant, the margins of France enter a constant plateau, while Austria’s margins successively jump to three lower levels and remain constant in the respective price ranges. On the other hand, the log wholesale margins of Slovakia, Germany, Portugal and Belgium show trends that cannot be adequately described as linear. On the other hand, the log margins of Spain change from a constant level with ${b}_{i}=0$ to a straight line with a negative slope, a behavior also exhibited by the log margins of Austria, France and Germany in the price range starting from ${log}_{10}\left({price}_{i}\right)=2.3 \left(\approx \EUR 200\right)$.

#### Linear mixed model

Overall, the log margin-log price graph shows sufficient linear relationships to assume a power distribution to wholesale margins and to estimate factor $${a}_{i}$$ and exponent $${b}_{i}$$ using Eq. 1. The linear mixed model with $${log}_{10}\left({price}_{i}\right)$$ as independent variable and country-specific random effects for intercept $${log}_{10}\left({a}_{i}\right)$$ and slope $${b}_{i}$$ lead to the estimates for $${a}_{i}$$ and $${b}_{i}$$ in Table 2. The Table shows the country-specific differences, first in the initial level of wholesale margins (intercept $${a}_{i}$$) and also with regard to the function progression (exponent $${b}_{i})$$. For all the countries listed, the overall trend is negative (regressive), albeit to varying degrees—less pronounced in Greece and Slovakia, but much stronger for Spain, Austria and France.

**Table 2:** Point estimates for country-specific ${a}_{i}$ and exponent ${b}_{i}$ of the power distribution

**Country**	${{\varvec{a}}}_{{\varvec{i}}}$	${{\varvec{b}}}_{{\varvec{i}}}$
Austria	4.71	-0.79
Belgium	0.08	-0.26
France	2.94	-0.69
Germany	0.64	-0.48
Greece	0.05	-0.15
Portugal	0.13	-0.29
Slovakia	0.07	-0.16
Spain	3.70	-0.91

**Fig. 5:**
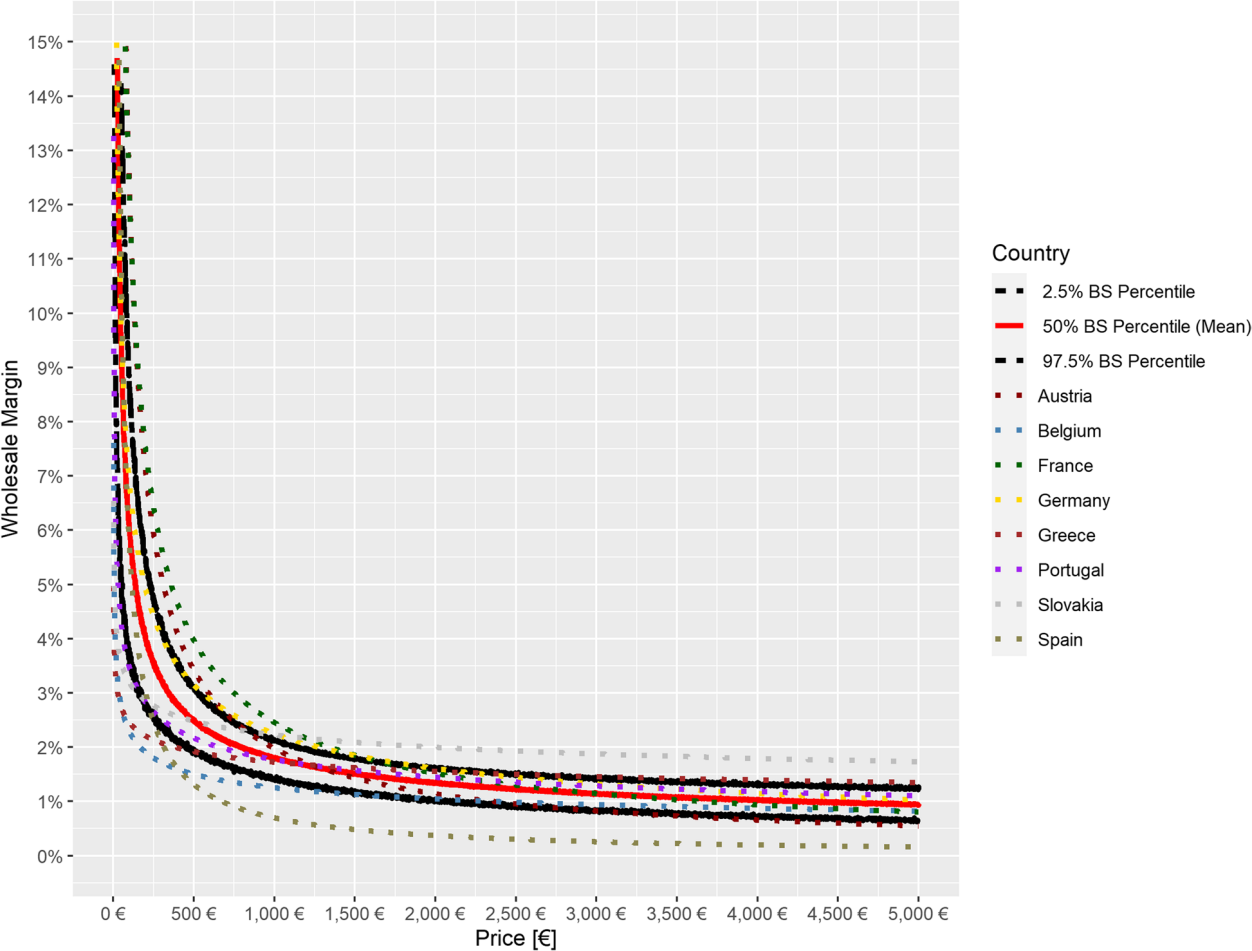
Estimated wholesale margins by price, selected European countries, overall estimated margins with 95% prediction interval. BS: bootstrap

Fitted wholesale margins for each country as well as the 2.5%, 50% and 97.5% percentiles of the bootstrap distributions are represented in Fig. 5. It can be shown that the approximately accurate mean estimate predicts the margins and the estimation interval includes most of the regulated countries’ margins.

As a reference point, margins (based on the model) are shown in Table 3 as a function of various prices.

**Table 3:** Estimated wholesale margins resulting from power distribution^a^

**Pharmacy purchase price**	**Estimated wholesale margin**
€5.00	66.73%
€10.00	34.26%
€25.00	16.23%
€50.00	9.87%
€75.00	7.52%
€100.00	6.26%
€250.00	3.64%
€500.00	2.52%
€750.00	2.07%
€1,000.00	1.81%
€1,500.00	1.51%
€2,000.00	1.34%
€2,500.00	1.23%
€3,000.00	1.14%
€3,500.00	1.08%
€4,000.00	1.03%
€4,500.00	0.99%
€5,000.00	0.95%

^a^Due to country-specific level jumps, the power distribution describes the margin trends of some countries less precisely in the lower price ranges. However, this lack of precision is accepted in favor of a uniform model type for the modeling

### Estimation quality

Original and estimated wholesale margins are compared and judged using MSE, which is generally defined as $\frac{\sum {\left(observed-fitted\right)}^{2}}{n}$. The lower the MSE, the better the fit. Furthermore, MSE is used to compare and judge estimated country-specific and overall margins with the uniform average margins provided by Kanavos [6] that might be used in price negotiations. As stated in the literature review, these averages are outdated. They are nevertheless used here for comparison with the results of the model, as there are no recent publications of average margins and the information published by Kanavos et al. [6] for the confidential countries is still currently used for estimates.

Since the denominator $n$ is the number of prices entering the estimating equation $\left(n=\mathrm{5,000}\right)$ and because its size makes the MSE numerically very small, thus making comparisons difficult, it is set to ‘1’ without affecting validity. In the final analysis step, the model’s estimate is compared with the estimate of uniform average margins.

MSE for each country between original wholesale margins and the overall estimated wholesale margins (50% bootstrap percentile), and between original margins and the average margins according to Kanavos et al. [6], respectively, are depicted in Table 4. It is obvious that the estimation with the model fits the original (true) margins much better (lower MSE) than the uniform average margins.

**Table 4:** MSE in comparison, according to the model vs. Kanavos et al. [6]

**Country**	**MSE according to the model (50% bootstrap percentile)**	**MSE according to Kanavos**
Austria	3.69	44.70
Belgium	2.90	33.12
France	4.13	11.74
Germany	1.96	6.98
Greece	4.52	3.42
Portugal	3.15	17.25
Slovakia	3.76	-
Spain	4.31	5.02

Based on these results for estimation in regulated countries, it is reasonable to assume that uniform averages bias estimates of margins for low- and high-priced drugs, even in countries with confidential wholesale margins. The potential magnitude of the bias is demonstrated below through impact calculations for typical cases.

### Impact analysis (cases)

The level of the estimated wholesale margins influences the level of the EU reference price of patent drugs, which is calculated using the actual real net prices in the relevant countries, adjusted by purchasing power parities and weighted by the corresponding country-specific sales. Questioning the use of uniform average margins, the issue of the potential effect of the presented alternative approach on the calculation of the EU reference price consequently arises. This effect varies—depending on the level of the actual dispensing prices in the relevant countries and on the number of countries in which the corresponding drug is available. To demonstrate the impact of using uniform average margins versus margins resulting from the statistical model on the EU reference price, a scenario representing the situation of newly launched products is created. According to the different country-specific market access and reimbursement procedures, there are usually certain countries in which a product is available earlier than in others. These countries are typically Denmark, Finland, the Netherlands and Sweden, which are therefore included in the following scenario [24].

Four differently priced fictitious drugs are considered in order to obtain a comprehensive impression of the effect across several price ranges. The PPPs were set at €500.00, €1,500.00, €3,500.00 and €4,000.00 in all the countries considered so as to ensure comparability. Based on these prices, the respective EU reference prices are calculated with regard to the requirements of the legal framework for its calculation—including the deduction of the uniform average margins from the literature, on the one hand, and the margins resulting from the model presented, on the other hand. As Kanavos et al. only states margin ranges for some countries instead of specific margins, the estimated average margins from the Swiss regulation are used for comparing the impact on the EU reference price. Afterwards, the prices were weighted by country-specific purchasing power parities and—for reasons of comparability—by population instead of country-specific sales data (would have to be estimated and so could cause a bias). Information on the required data used to calculate the EU reference price is provided (Additional file 2). The resulting absolute and percentage differences between the EU reference prices are shown in Table 5. With regard to high-price drugs in particular, those differences in margins can lead to changes of the European reference price of more than €100, meaning that the EU reference price is 3.73% lower when using uniform average margins instead of using the margins resulting from the model.

**Table 5:** Launch scenario Denmark, Finland, Netherlands, Sweden

**Pharmacy purchase price**	**Results from statistical model**		**Results from literature (e.g. Swiss Regulation **[13]**)**	**Difference EU reference price (%)**
	**Margin**	**EU reference price**	**EU reference price using average margins (NL: 6.5%; FI: 3%; DK: 6.5%; SWE: 2.7%)**	
€500.00	2.52%	€448.16	€437.58	-2.36%
€1,500.00	1.51%	€1,353.31	€1,312.73	-3.00%
€3,500.00	1.08%	€3,181.79	€3,063.03	-3.73%
€4,000.00	1.03%	€3,638.13	€3,500.60	-3.78%

It can be concluded that the use of uniform average margins might have a significant negative influence on EU reference prices and therefore also on the reimbursement prices negotiated in the area of patent-protected medicines.

## Discussion

Estimation procedures of confidential wholesale margins have not been discussed in the literature so far. Published estimates of margins for individual drugs of a specific price level often refer to the difference between average margins and PPP. In most European countries, wholesale price regulations provide that a margin regresses with increasing price. In combination with left-skewed volume distributions (high proportion of low-priced drugs (generics), lower proportion of high-priced drugs) for these countries with transparent margins, calculating the EFP using uniform average margins would have a distorting effect on much of the price spectrum. This distorting effect is amplified by the fact that data on the level of average margins are rarely (irregularly) published.

Based on this finding, it seems plausible to conclude that there would also be a distorting effect in countries with confidential wholesale margins if the uniform average margin was used to calculate an EFP. In particular, the expert interviews with wholesalers in Denmark, Finland, Sweden and the Netherlands confirmed that wholesaler margins generally behave in principle regressive to rising prices. By conducting the impact analyses, it could be shown that price negotiations in Germany can strongly be influenced depending on whether uniform average margins from the literature or margins that correspond to our model are used. The effect could vary, when (a) real country-specific sales data are used for weighting instead of population data (which have not been used in this case for reasons of comparability), e.g. if a country with confidential margins reports disproportionately high sales and would be therefore weighted more strongly, and (b) if there are fewer countries in which the product is available.

Comparable effects could also be assumed with regard to price negotiations in other countries in which confidential wholesale margins need to be estimated, too. While previous literature stated that the best method for estimating confidential wholesale margins was to use average margins, the present model extended the low level of research substantially.

## Conclusions

Special characteristics of the pharmaceutical market with a very wide range of drug prices and at the same time nearly similar distribution costs of drug packages highlight the necessity to question uniform percentages to estimate wholesale margins for differently priced pharmaceuticals. Based on the known margins and margin patterns determined by regulation, a statistical model was created that shows how percentage margins of wholesalers develop regressively as drug prices rise. It could also be shown that the model allows a significantly better estimate of wholesale margins for different prices. For countries with confidential wholesale margins, estimation using the shown or a similar regressive function is preferable to estimation using average margins. This is supported on the one hand by general considerations, according to which uniform percentage margins for expensive drugs lead to high absolute amounts, which cannot be substantiated by corresponding cost differences in wholesale. On the other hand, expert interviews with wholesalers and a pharmaceutical manufacturer from countries with confidential wholesale margins confirmed that wholesale margins are in principle also regressive to rising prices. Therefore, it is reasonable to assume that using literature-based uniform (i.e. price-independent) average margins for countries with confidential margins, as practiced, does not lead to realistic estimates. This paper provides an alternative estimation approach for this purpose, which determines country-specific price-margin functions for the countries with transparent margins and derives an ‘average’ estimation function on this basis. This approach assumes regressive margin developments to the same extent as they exist in real terms taking the average function for countries with transparent margins. Even if the actual margin development in these countries deviates from this, it is reasonable to assume that—especially in the case of high-price products—significantly lower estimation errors are achieved compared to the use of uniform, price-independent average margins. Accordingly, it is advisable to estimate EFP in countries with confidential wholesale margins based on a price-margin function similar to the methodology proposed here rather than using uniform average margins.

## Limitations

One key assumption of the modeling is the regressivity of wholesale margins in the European countries with confidential wholesale margins. Only one interviewee could be obtained for each country to verify this assumption. Even if there are only a few wholesalers in some of the countries and the interviewees were in strategically relevant positions for the assessment, the assumption is based on the statements of individual experts. Overall, the confidentiality of margins limits the survey of pricing principles in these countries.

### Supplementary Information

**Additional file 1.** Regulations of wholesale margins by country.

**Additional file 2.** Expert Interview guideline.

## Data Availability

Datasets analyzed during the current study are available from the corresponding author on reasonable request.

## Abbreviations

AMNOG: German Act to Reorganize the Pharmaceuticals’ Market in the Statutory Health Insurance System
BS: Bootstrap
DDD: Defined Daily Doses
EFP: Ex-Factory Price
EFPIA: European Federation of Pharmaceutical Industries and Associations
EU: European Union
MSE: Mean Squared Error
PPP: Pharmacy Purchase Price
SHI: Association of Statutory Health Insurers

## Footnotes

1 Although the United Kingdom is still part of the EU reference basket, it no longer fulfills the requirements of the framework regulation following its withdrawal from the European Union and is therefore not considered in the context of this publication. Nevertheless, the reflection of UK in the basket does not change neither the method described in this paper nor the results for the other countries and the statistical assumptions could be applied analogously to the UK.

2 In the Netherlands, several factors determine whether wholesale margins have to be estimated in the context of price negotiations for innovative patent-protected medicines. In case of direct delivery and use in hospitals wholesale services are not required, so the ministry's published maximum prices can be interpreted as ex-factory prices [4]. For medicines covered through the retail reimbursement system, reimbursement is regulated on the level of official pharmacy purchasing prices, so that the confidential wholesale margins must be estimated.

3 In the following, wholesale margins are understood to be the gross profits that wholesalers receive as remuneration for their services. They can be defined as percentage mark-ups on the EFP or as fixed, absolute prices, also known as ‘wholesale mark-up’.
