# Supplementary material for: Examining confidential wholesale margin estimates in European countries for the price negotiation of patented drugs in Germany: a statistical model
Source: Health Econ Rev. 2024 Apr 12;14:27. doi: 10.1186/s13561-024-00503-9 (PMC11010283; doi:10.1186/s13561-024-00503-9)
Supplement: Supplementary file 1 — Additional file 1. Regulations of wholesale margins by country. [file 13561_2024_503_MOESM1_ESM.docx]

## Additional file 1

**Regulations of wholesale margins by country**

**Table A1 Country-specific margin regulations**

| **Country** | **Calculation of the wholesale margin according to relevant price ranges** |
| --- | --- |
| Belgium [16] | €0 < EFP < €2.33: €0.35  €2.33 ≤ EFP ≤ €15.33: 15.00% of EFP  EFP > €15.33: €2.00 + 0.9% of (EFP - €15.33) |
| Germany [17] | €0 < EFP ≤ €1,200: 3.15% of EFP + €0.70  EFP > €1,200: €37.80 + €0.70 |
| France [18] | €0 < EFP < €4.50: €0.30  €4.50 ≤ EFP ≤ €468.97: 6.93% of EFP  EFP > €468.97: €32.50 |
| Greece [19] | €0 < EFP ≤ €200: 4.9% of EFP  EFP > €200: 1.5% of EFP |
| Austria [20] | €0 < EFP ≤ €6.06: 15.5% of EFP  €6.07 < EFP ≤ €6.22: EFP + max. €7  €6.23 ≤ EFP ≤ €12.11: 12.5% of EFP  €12.12 < EFP ≤ €12.32: EFP + max. €13.62  €12.33 ≤ EFP ≤ €53.78: 10.5% of EFP  €53.79 < EFP ≤ €54.77: EFP + max. €59.43  €54.78 ≤ EFP ≤ €181.68: 8.5% of EFP  €181.69 < EFP ≤ €184.22: EFP + max. €197.12  €184.23 ≤ EFP ≤ €339.14: 7% of EFP  EFP ≥ €339.15: €23.74 |
| Portugal [21] | €0 < EFP ≤ €5: €0.25 + 2.24% of EFP  €5 < EFP ≤ €7: €0.52 + 2.17% of EFP  €7 < EFP ≤ €10: €0.71 + 2.12% of EFP  €10 < EFP ≤ €20: €1.12 + 2.00% of EFP  €20 < EFP ≤ €50: €2.20 + 1.84% of EFP  EFP > €50: €3.68 + 1.18% of EFP |
| Slovakia [22] | €0 < EFP ≤ €2.66: 14.10% of EFP  €2.67 < EFP ≤ €5.31: 11.10% of (EFP – €2.66) + €0.37  €5.32 < EFP ≤ €7.97: 8.10% of (EFP – €5.31) + €0.67  €7.98 < EFP ≤ €13.28: 5.10% of (EFP – €7.97) + €0.88  €13.29 < EFP ≤ €23.24: 3.30% of (EFP – €13.28) + €1.16  €23.25 < EFP ≤ €39.83: 2.70% of (EFP – €23.24) + €1.48  €39.84 < EFP ≤ €73.03: 2.40% of (EFP – €39.83) + €1.93  €73.04 < EFP ≤ €165.97: 2.25% of (EFP – €73.03) + €2.73  €165.98 < EFP ≤ €331.94: 2.10% of (EFP – €165.97) + €4.82  €331.95 < EFP ≤ €663.88: 1.95% of (EFP – €331.94) + €8.31  EFP > €663.88: 1.80% of (EFP – €663.88) + €14.78 |
| Spain [23] | €0 < EFP ≤ €91.63: 7.6% of EFP  EFP > €91.63: €7.54 |

**Table A2** **Data used to calculate European reference price. Source: Eurostat. (Effective 01.09.2022)**

| **Country** | **Population Size** | **Purchasing Power Parity** | **Exchange Rate** |
| --- | --- | --- | --- |
| Denmark | 5.840.045 | 9,88 | 7,4392 |
| Finland | 5.533.793 | 1,24 | - |
| Netherlands | 17.475.415 | 1,15 | - |
| Sweden | 10.379.295 | 13,05 | 10,73 |
