# Supplementary material for: Examining confidential wholesale margin estimates in European countries for the price negotiation of patented drugs in Germany: a statistical model
Source: Health Econ Rev. 2024 Apr 12;14:27. doi: 10.1186/s13561-024-00503-9 (PMC11010283; doi:10.1186/s13561-024-00503-9)
Supplement: Supplementary file 2 — Additional file 2. Expert Interview guideline. [file 13561_2024_503_MOESM2_ESM.docx]

## Additional file 2

**Expert Interview guideline**

The key question at stake is the following:

- Is the price level – among other factors – taken into account in the WHS margin negotiations?
- Besides other factors that are taken into account when negotiating margins, do higher prices (usually) lead to lower %-margins/lower prices lead to higher %-margins?
- In your country, are high-priced (Rx) medicines mainly supplied by wholesalers or are there specific rules for high-priced medicines, e.g. that they are mainly delivered directly to hospitals? If so, are the medicinal products this rule applies to defined?

In the case of a fixed margin:

- How is it compiled? Is this possibly an average for many put-ups/the whole portfolio of a company? Does this average include quantities such that the impact of high-priced products can be considered in the average? Does it play a role if the portfolio includes generic or patented/regulatory protected drugs?
- Are there negotiations? Which factors are considered? Are high-priced drugs possibly accompanied by a lower margin (within the average fixed margin)?
